# Overweight and lower age at menarche: evidence from the Italian HBSC cross-sectional survey

**DOI:** 10.1186/s12905-018-0659-0

**Published:** 2018-10-19

**Authors:** Giacomo Lazzeri, Claudia Tosti, Andrea Pammolli, Gianmarco Troiano, Alessio Vieno, Natale Canale, Paola Dalmasso, Patrizia Lemma, Alberto Borraccino, Felice Petraglia, Stefano Luisi

**Affiliations:** 1 Department of Molecular and Developmental Medicine University of Siena, Siena, Italy; 2 Department of Developmental and Social Psychology, University of Padova, Padova, Italy; 3 Department of Public Health and Paediatrics, University of Turin, Torino, Italy; 4 Department of Biomedical, Experimental and Clinical Sciences “Mario Serio”, Careggi University Hospital, University of Florence, Florence, Italy; 5 CREPS-Research for Health Education and Promotion, University of Siena, Siena, Italy

**Keywords:** Early puberty, BMI, Adolescent

## Abstract

**Background:**

A unique standardized national dataset on adolescent girls (21 regions) participating in the Italian Health Behaviour in School-aged Children Study (HBSC) was used to investigate the correlation between body mass index (BMI) and age at menarche.

**Methods:**

Two independent nationally representative survey datasets: one on 15-year-olds (*n* = 6907, in 21 regions, year 2013/2014) and one on 11-year-olds (*n* = 10,128, in 20 regions, year 2009/2010) were analysed. The survey instrument was a self-report questionnaire. Median age at menarche and 95% confidence intervals (CIs) were estimated by means of Kaplan–Meier analysis. Hierarchical models were used to assess the relationship between BMI and age at menarche (months). “Region-level obesity” was measured as the prevalence of overweight/obesity (%) in each region.

**Results:**

Region-level median age at menarche ranged between 12 years/3 months and 13 years/4 months. Region-level prevalence of overweight among 15-year-old girls ranged between 4 and 19%. Age at menarche was inversely associated with individual BMI (unstandardized regression coefficient beta = − 0.70; 95% CI, − 0.84 to − 0.56). Individual- and class-level measures of BMI accounted for 50% of the region-level variance in age at menarche.

**Conclusions:**

The results show that overweight in childhood is in relation with the early puberty in girls. Future surveys may take into account this report to clarify if overweight is the cause or consequence of early menarche.

## Background

Over the past 30 years, a steady increase in the prevalence of overweight has been observed in all age groups from children to adults all over the world, including high-income and low- to middle-income countries [1]. Thus, given the serious health consequences associated with overweight and obesity, new public health challenges have been developed globally. Specifically, the consequences of overweight include insulin resistance and diabetes mellitus, particularly resistance to leptin and insulin, two hormones that play an important role in the time of puberty and its progression and have an impact on the risk of obesity [2]. We must also take into account other non-communicable diseases such as hypertension, stroke and ischemic heart disease, musculoskeletal and orthopaedic disorders, early onset of sexual behaviour and use of substances, mental health problems (body dissatisfaction and eating disorders and depression) and some forms of cancer [3–5]. The impact of these diseases both on the public health and economic health management level is of considerable importance [6]. Obesity must be addressed early in life; however, the current approaches towards effective treatment and prevention of childhood obesity remain far from satisfactory [7, 8]. Hence, the prevalence of obesity continues to increase in a lot of populations around the world. The Commission on Ending Childhood Obesity of the World Health Organisation concluded that no single intervention could halt the rise of the obesity epidemic and recommended that attention should be devoted to preventive measures in three sensitive periods of life: preconception and pregnancy, infancy and early childhood, and older childhood and adolescence [9].

In early adolescence, obesity has an important impact on health leading to earlier pubertal onset, manifested by earlier thelarche [10] and menarche [11]. There are many theories that explain why the trend towards earlier puberty has occurred, ranging from environmental toxins to changes in socioeconomic status, but it is clear that nutritional status plays an essential role in the timing and progression of puberty. However, it is yet to be elucidated whether weight gain precedes early puberty, early puberty predisposes to abnormal weight gain or both.

Several epidemiological reports in the past 30 years indicated a relationship between earlier menarche in girls and increased body mass index (BMI) [12]. Increased body fat at birth or in early childhood and a rapid increase in BMI during infancy predict earlier onset of puberty [13–15]. However, there is a lack of standardised comparable data to evaluate the role of overweight (or BMI) in the affecting age at menarche across Italian regions.

The present study is the first to examine the relationship between obesity and age at menarche by utilising standardised data from all Italian regions, without the problems of interpretation that arise from different methodologies and different cohorts.

## Methods

We conducted a cross-sectional study based on the Italian “Health Behaviour in School-aged Children” (HBSC) study, and carried out according to the international HBSC protocol. The HBSC is a collaborative cross-national WHO survey that collects data on health behaviour every 4 years on nationally representative samples of adolescents aged 11–13-15 years, using “school class” as the primary sampling unit. A systematic cluster sampling using probability proportional to population size (PPS) was used to have a nationally representative sample. The teachers of the classes included in the sample were informed and asked to deliver an information sheet, containing basic information about the survey, to each student, who in turn had to give it to parents or guardians and an opt-out form to be signed and sent back only in case of refusal to participate. The Italian study protocol and the questionnaire were approved by the Ethics Committee of the University of Turin. Data were collected anonymously, and standard protection measures were ensured to preserve confidentiality. A standardized, anonymous, self-compiled questionnaire was administered by trained teachers to students. A detailed description of the aims, theoretical framework and protocol of the international and Italian studies can be found elsewhere [16–18]. In the 2013/2014 survey, school/class- and pupil-level response rates exceeded 85% in most regions [19]. A nationally representative random samples of 15-year-old girls (*n* = 6907), was used to have internationally comparable data on age at menarche and BMI. Girls came from 906 schools in the 21 regions that participated in the 2013/2014 HBSC Italian survey. Sample sizes ranged from 150 (Valle d’Aosta) to 774 (Veneto). Furthermore, aggregated region-level data on BMI among 11-year-old students (*n* = 10,128) from 1160 schools coming from 20 of these regions, which had also participated in the 2009/2010 HBSC Italian survey [18], were used. Sample sizes ranged from 281 (Valle d’Aosta) to 763 (Piedmont).

## Measures

The Menarcheal status and information about the age of menarche onset were obtained through the question “Have you begun to menstruate?” whose answers could be “No, I have not yet begun to menstruate” or “Yes, I began at the age of __years and _ months”. Girls were divided in two categories as pre- or post-menarcheal. Most of the girls were excluded from our analysis because of the lack of age at menarche, specifically 2424 girls (35.6%).

Self-reported height and weight were used to calculate the Body mass index (kg/m^2^). According to the WHO age- and sex-specific cut-offs [20] students were categorized as overweight or obese (hereafter indicated to as “overweight”).

The aggregate regional prevalence of overweight for the 15 years in the 2013/2014 survey was calculated. The same prevalence was calculated for 11 years in the 2009/2010 survey so as to provide an independent measure of the overweight level at regional level. Furthermore, this group is based on a predominantly pre-menarcheal sample (78.3% pre-menarcheal) drawn from the same population cohort that was sampled again in 2013/2014 when it aged 15 years.

### Socioeconomic status

Socioeconomic status (SES) was evaluated through the Family Affluence Scale (FAS) [21]. The FAS is a validated measure of material affluence that assigns a composite score on four items: number of family-owned computers (none, one, two or more); having own bedroom (yes/no); number of cars in family (none, one, two or more); holidays with family in the last year (none, once, twice or more). The sum of responses produces an ordinal scale ranging from 0 to 7. The measure is split into approximate tertiles of family affluence across all regions [22]. In 10% of girls (*n* = 428) with complete data for age at menarche and BMI, FAS data were missing.

### Statistical analyses

Age (at menarche), which is a continuous variable, was expressed as a median value with its 95% confidence interval (CI) by means of the Kaplan-Meier method. A linear regression model was used to assess the relationship between BMI and age (at menarche): age (at menarche) represented the dependent variable and it was adjusted for age and FAS among subjects aged 15 years in 2013/2014. Another regression model was used to calculate the relationship between region-level median age (at menarche) and prevalence of overweight among 15 year-old subjects in 2013/2014 and 11 year-old subjects in 2009/2010. The relationship between BMI and age (at menarche) at the individual and region levels was finally assessed through hierarchical models at four levels (individual, school, sampling strata (north, centre, south) and region.

This analysis was restricted to 20 regions that furnished these data.

- Model 1 included only age and family affluence.
- Model 2 also included individual BMI.
- Model 3 included aggregate prevalence of overweight among 11 year-olds in 2009/2010.

The region-level intraclass correlation (ICC), which measures the proportion of the variance in age (at menarche) that is attributable to the region-level, was used. The ICC from Model 2 and Model 3 are expressed as a percentage of the ICC from Model 1, in order to show the change in region-level residual variance. Age (at menarche) was imputed as current age for those who were premenarcheal. All analyses were carried out using the software SPSS 22.0 and adjusted for sampling design by means of the Complex Samples module.

## Results

The mean age of 15-year-olds in the 2013/2014 survey was 15.9 years (SD = 0.31), and that of 11-year-olds in the 2009/2010 survey was 11.4 years (SD = 0.31). Among 15-year-old girls with complete menarche data, 2.2% were pre-menarcheal, and the mean FAS score was 4.6 (SD = 1.27).

Remarkable differences in the age at menarche were observed among individuals and across regions in the sample of 15-year-old surveyed in 2013/2014. In 95% of individuals, age (at menarche) ranged between 10 years 0 months and 14 years 7 months. No subject was younger than 10 years 0 months. The region-level median age (at menarche) ranged from 12 years and 3 months in Lazio to 13 years and 4 months in Bolzano. The region-level prevalence of overweight varied across regions: from 4% (Bolzano and Piedmont) to 19% (Campania and Puglia) among 15-year-olds in 2013/2014, and from 10% (Bolzano) to 35% (Campania) among 11-year-olds in the 2009/2010 survey (Table 1).

**Table 1:** Summary by region of median age at menarche, and percentage overweight/obese at age 15 and at age 11. HBSC study, 2013/2014 and 2009/2010

Region	15-year-old girls (2013/2014)				11-year-old girls (2009/2010)
	Median age at menarche			Percentage	Percentage
	*N*	Months (years and months)	95% CI (months)	overweight/obese^a^	overweight/obese^b^
Abruzzo	152	154 (12 years 10 months)	(151.50–156.50)	14.1	24.9
Basilicata	126	151 (12 years 7 months)	(148.59–153.42)	13.8	23.0
Bolzano	214	160 (13 years 4 months)	(157.79–162.21)	4.2	9.7
Calabria	194	149 (12 years 5 months)	(147.83–150.18)	12.3	25.9
Campania	153	150 (12 years 6 months)	(147.80–152.20)	18.8	34.7
Emilia R.	214	154 (12 years 10 months)	(151.13–156.87)	11.2	19.2
Friuli V.G.	241	154 (12 years 10 months)	(152.07–155.94)	8.2	19.9
Lazio	164	147 (12 years 3 months)	(144.61–149.39)	11.8	19.8
Liguria	272	152 (12 years 8 months)	(150.05–153.95)	9.7	15.1
Lombardy	288	155 (12 years 11 months)	(152.64–157.36)	5.8	18.8
Marche	283	156 (13 years 0 months)	(154.10–157.90)	11.0	23.8
Molise	172	152 (12 years 8 months)	(148.96–155.04)	8.5	24.5
Piedmont	195	153 (12 years 9 months)	(149.98–156.02)	4.2	16.3
Puglia	204	150 (12 years 6 months)	(147.86–152.14)	19.1	29.4
Sardinia	121	152 (12 years 8 months)	(149.87–154.13)	8.8	16.1
Sicily	195	149 (12 years 5 months)	(147.00–151.00)	15.2	25.4
Tuscany	260	150 (12 years 6 months)	(148.22–151.79)	6.5	16.5
Trento	230	154 (12 years 10 months)	(151.26–156.74)	7.7	14.0
Umbria	222	153 (12 years 9 months)	(151.46–154.55)	9.6	16.3
Valle d’Aosta	98	158 (13 years 2 months)	(154.84–161.16)	6.5	16.2
Veneto	485	156 (13 years 0 months)	(154.32–157.68)	7.3	No data
All regions	4483	153 (12 years 9 months)	(152.42–153.58)	12.2	23.9

^a^Total sample size 4259

^b^Total sample size 6535

As shown in Table 2, significant negative association between individual BMI and age (at menarche) was observed on 15-year-old sample (coming from 21 regions). The unitary increase of BMI led to a 1 month earlier onset of menarche (β = − 0.76; 95% CI, − 0.89 to − 0.63).

**Table 2:** Linear regression analysis for age at menarche (months) among 15-year-olds

Independent variables	Unstandardized regression coefficient	95% CI
Body mass index (BMI)	−0.76	−0.89 to − 0.63
Current age (months)	0.72	0.62 to 0.82
FAS score	0.07	−0.22 to 0.37
Constant	31.85	12.82 to 50.88

*N* = 3767, *p* < 0.001, age at menarche and current age are measured in months

*CI*confident interval

Data source: HBSC 2013/2014 survey

Also considering the single regions, menarche occurred earlier in girls with a higher BMI (β coefficients ranged from − 1.40 to − 0.30); however, in four regions, namely Molise (β = − 0.52), Sardinia (β = − 0.22), Umbria (β = − 0.12) and Valle d’Aosta (β = − 0.79), this relationship was not statistically significant.

A remarkable negative association was observed between aggregate prevalence of overweight and median age (at menarche) among 15-year-olds in 2013/2014 (Table 3) by treating the regions as units. The median age (at menarche) was approximately 10 days lower (β = − 0.33; 95% CI, − 0.50 to − 0.15), for each percentage of increase in the region-level prevalence of overweight. As the region-level prevalence of overweight ranged between 4 and 19%, the maximum difference observed in the age (at menarche) was 24 weeks (approximately 6 months). When the analysis was repeated on region-level data on overweight status among 11-year-olds in 2009/2010, a different relationship emerged (β = 0.006; 95% CI, − 0.19 to 0.21).

**Table 3:** Linear regression analysis for regional-level median age at menarche among 15-year-olds in 2013/2014

Model		Unstandardized regression coefficient	95% CI
1. 15-year-old girls 2013/2014	Region-level prevalence of overweight (%)	−0.33	− 0.50 to − 0.15
	Constant	155.18	152.55 to 157.82
2. 11-year-old girls 2009/2010	Region-level prevalence of overweight (%)	0.006	−0.19 to 0.21
	Constant	131.22	124.94 to 137.49

Region-level median age at menarche is measured in months; region-level of overweight is measured in percent

Model 1: Independent variable: prevalence of overweight among 15-year-olds (2013/2014), *N* = 21 regions, *p* = 0.001

Model 2: Independent variable: prevalence of overweight among 11-year-olds (2009/2010), *N* = 19 regions, *p* = 0.95

*CI* confidence interval

Data source: HBSC 2013/2014, HBSC 2009/2010

To evaluate the association between BMI and age (at menarche) multilevel regression models were used. No significant differences in age (at menarche) emerged among the regions; only the coefficient of current age differed significantly (Table 4: Model 1). In the second model, in addition to the significant difference in the coefficient of current age, region-level variance in age (at menarche) increased the ICC by 6.8%, and for each unit increase in BMI, age (at menarche) was approximately, and significantly, 1 month lower (Table 4: Model 2). In the third model, the fixed-effect estimates did not change, but accounted for a further 60% of region-level variance in age (at menarche) (Table 4: Model 3). In this final model, for each unit increase in BMI at the individual level, menarche occurred approximately 1 month earlier (β = − 0.70; 95%CI, − 0.84 to − 0.56). In all three models, the school-level coefficient proved significant; thus, age (at menarche) varied with BMI, but in a different way among schools. This variation was not significantly different among regions, nor at the strata level (north, centre and south).

**Table 4:** Summary of results from multilevel models of age at menarche (months) among 15-year-olds across 21 regions

Factors	Model 1 adjusted for structural covariates	Model 2 addition of individual BMI	Model 3 addition of region-level overweight
Fixed effects (95% CI)			
Individual level			
Family affluence	−0.03 (− 0.37 to 0.30)	−0.12 (− 0.45 to 0.21)	−0.12 (− 0.45 to 0.21)
Current age (months)	0.53 (0.41 to 0.66)^*^	0.53 (0.41 to 0.64)^*^	0.53 (0.41 to 0.65)^*^
Body mass index (BMI)	–	−0.70 (− 0.84 to − 0.56)^*^	−0.70 (− 0.84 to − 0.56)^*^
Region level			
Prevalence of overweight (%) of 11-year-olds in 2009/2010	–	–	0.04 (− 0.11 to 0.20)
Random effects (SE)			
Region-level variance	0.25 (0.58)	0.26 (0.56)	0.16 (0.72)
Region-level ICC as percentage of model 1 ICC^a^	100%	106.8%	47%
Strata-level variance (north-centre-south)	1.33 (1.68)	0.68 (1.03)	1.45 (2.66)
Class-level variance	18.99 (2.68)^*^	18.11 (2.61)*	18.11 (2.61)*
Residual (individual level) variance	6069.80 (155.56)	5906.23 (151.23)	5907.08 (151.58)
Log likelihood	−15,423.05	− 15,375.21	− 15,376.80
Likelihood-ratio test for change in log likelihood		p < 0.001	*p* < 0.01

Age at menarche is measured in self-report age in months, or current age for premenarcheal girls

Model 1: Adjusted only for current age and family affluence

Model 2: Adjusted for individual BMI measure in addition to current age and family affluence

Model 3: Adjusted for region-level prevalence of overweight (%) among 11-year-olds in 2009/2010, in addition to individual-level BMI, current age, and family affluence

*CI* confidence interval

^a^Region-level intraclass correlation coefficient measures the proportion of observed variation in age at menarche attributable to region level

*p < 0.05 significant result for Fixed or Random Effect

## Discussion

Since the 1980s, several epidemiological studies have indicated an association between early puberty (particularly in girls) and increased BMI, which is an index that remains at the epidemiological level and is mostly used for measuring, even if indirectly, overweight and obesity [23–25]. Our work, through data collected with a standardised methodology, contributes to providing updated data on the association between adolescent obesity and age at menarche across Italian regions.

As already highlighted by Currie et al., between the countries in Europe and North America [26, 27], the average age at menarche differed by at least 1 year even among Italian regions.

In recent years, the rate of decline in menarche age in industrialised countries varies from country to country, and this may be related to the change in the prevalence of obesity [25]. A study conducted in the United States, which investigated the changes in the age of menarche, suggested the existence of a direct relationship between the decline of age at menarche and being overweight [28]. Our work also contributes to improving knowledge on this assumption.

Consistent with the findings of the previous 2009/10 HBSC Italian survey [19], also in the 2013/14 survey, there was a fivefold difference in the prevalence of overweight among 15-year-old girls in 21 Italian regions.

Higher rates of overweight were found in central and southern Italy, and lower rates were found among the northern regions. Notably, lower median ages at menarche were generally observed in the southern regions, where the prevalence of overweight is higher. Data collected in 2014 showed a decrease in the prevalence of overweight/obesity in all age groups in all regions in comparison with the previous survey of 2010. There was a North–South gradient among the regions, with the highest prevalence of overweight in the southern regions. Specifically, the highest percentages were found in the Campania region, where 5.8% were obese and 21.4% were overweight. The lowest obesity values (1.1%) were found in the PA of Bolzano, in Lombardy and in the PA of Trento; the PA of Bolzano also had the lowest percentage (7.7%) of overweight young people. The HBSC data of the Italian survey showed that three-quarters of the regions participating in both the 2009/10 and 2013/14 surveys displayed a slight decrease in the prevalence of overweight between survey sweeps. However, the ‘overweight risk’ was high and differed from north to south. In Italy, the median age at menarche in 2005/06 was lower than that in the present study (12 years 5 months vs. 12 years 9 months). This does not correspond to what was found in the studies conducted in other industrialised countries [26, 29, 30], but it corresponds to the fact that the rate of decline varies between countries [25].

Marked changes in gonadal function occur in girls from the onset to the end of puberty. Specifically, before puberty, despite the low concentrations of oestrogen, the negative feedback mechanism is extremely powerful and suppresses the hypothalamus and the pituitary. During this period, the positive feedback mechanism is not active. During puberty, the negative feedback effect of oestrogen is attenuated, but other substances, such as inhibins, are incorporated into the system. Towards the end of puberty, the positive feedback mechanism becomes active and starts normally. Finally, after puberty, the two feedback mechanisms are the main determinants of the relationships between the ovaries and the hypothalamic–pituitary system [31].

Longitudinal studies indicate BMI as a risk factor for early puberty with overweight that predicts the onset of different markers [32]; this is in agreement with the inverse association between BMI and the age at menarche detected at the individual and class levels in our study.

In a study, Kaplowitz states that there is a direct association between leptin, gonadotropin and puberty times [12].

Although our article agrees with this hypothesis, other research indicates that early puberty is a cause of subsequent obesity [25]. Moreover, given the possibility shown by some authors of the possible influence of common genes on the association between menarche and overweight [28], it is necessary to study in more detail the contribution of genetic and environmental factors.

As changes in diet and physical activity also occur at this age, there is a ‘triple hit’ concerning obesity. Indeed, adolescence is the ‘perfect storm’ of inciting factors, which conspire to cause and promulgate the obese state. Girls are particularly susceptible because of increased insulin resistance due to oestrogen exposure.

This study has several limitations: (i) the cross-sectional nature of the study, (ii) the survey that was not originally designed for this particular study and (iii) the self-reported information. Furthermore, 2424 girls (35.6%) were excluded from the analyses; among them, most were excluded because the month of reported age at menarche was missing.

Despite these limitations, this study provided novel information about the timing of menarche through the analysis of nationally representative data on Italian girls. Besides, the girls who participated in the survey were close to the time of menarche, so it limits the memory bias, enhancing the value of these data.

Premenarche girls have been included in hierarchical models by equating their ages from menarche to the current one. This relatively conservative procedure involving few cases (3% of 15-year-old girls was premenarche) was adopted to avoid reinforcing the relationship between increasing BMI and decreasing age at the menarche. Furthermore, this procedure allowed us to maximise the number of girls involved in the analysis.

## Conclusions

Current age, individual and class (school) level of BMI were associated with variations in age at menarche, whereas region- and strata-level BMIs were not associated with age at menarche.

The results indicate a negative association between prevalence of overweight and median age at menarche among Italian 15-year-old girls at the regional level.

It would, therefore, be interesting to investigate how the overweight interacts with lifestyles, the environment and genetics, to better understand the class-level associations highlighted in our research.

## Abbreviations

BMI: Body Mass Index
FAS: Family Affluence Scale
HBSC: Health Behaviour in School-aged Children
N: Normal-weight
O: Obesity
Ow: Overweight
Ow/O: Overweight including Obesity
SES: Socioeconomic Status
U: underweight
WHO: World Health Organization
